# The Less Well-Known Little Brothers: The SLC9B/NHA Sodium Proton Exchanger Subfamily—Structure, Function, Regulation and Potential Drug-Target Approaches

**DOI:** 10.3389/fphys.2022.898508

**Published:** 2022-05-25

**Authors:** Manuel A. Anderegg, Gergely Gyimesi, Tin Manh Ho, Matthias A. Hediger, Daniel G. Fuster

**Affiliations:** ^1^ Department of Nephrology and Hypertension, Inselspital, Bern University Hospital, University of Bern, Bern, Switzerland; ^2^ Membrane Transport Discovery Lab, Department for BioMedical Research, University of Bern, Bern, Switzerland

**Keywords:** membrane transport, sodium proton exchange, SLC9B, NHA1, NHA2, NHE

## Abstract

The SLC9 gene family encodes Na^+^/H^+^ exchangers (NHEs), a group of membrane transport proteins critically involved in the regulation of cytoplasmic and organellar pH, cell volume, as well as systemic acid-base and volume homeostasis. NHEs of the SLC9A subfamily (NHE 1–9) are well-known for their roles in human physiology and disease. Much less is known about the two members of the SLC9B subfamily, NHA1 and NHA2, which share higher similarity to prokaryotic NHEs than the SLC9A paralogs. NHA2 (also known as SLC9B2) is ubiquitously expressed and has recently been shown to participate in renal blood pressure and electrolyte regulation, insulin secretion and systemic glucose homeostasis. In addition, NHA2 has been proposed to contribute to the pathogenesis of polycystic kidney disease, the most common inherited kidney disease in humans. NHA1 (also known as SLC9B1) is mainly expressed in testis and is important for sperm motility and thus male fertility, but has not been associated with human disease thus far. In this review, we present a summary of the structure, function and regulation of expression of the SLC9B subfamily members, focusing primarily on the better-studied SLC9B paralog, NHA2. Furthermore, we will review the potential of the SLC9B subfamily as drug targets.

## 1 Introduction

About 10% of the human genome encodes for transporter-related proteins, which substantiates the importance of the equilibrium between the intracellular and extracellular environment to maintain biological homeostasis (Hediger et al., 2013). There are four big groups of transporter proteins: ATP-binding cassette transporters (ABC), ATPase pumps, ion channels and ionotropic receptors, as well as solute carriers (SLCs) (Hediger et al., 2004; Hoglund et al., 2011). The SLC family includes passive transporters, ion transporters, and exchangers that mediate the flow of various substances such as sugars, amino acids, nucleotides, inorganic ions, and drugs across the cell membrane (Hediger et al., 2004; Hoglund et al., 2011; Hediger et al., 2013). Among them, Na^+^/H^+^ exchangers (NHEs) are a group of ion transporter proteins found in all phyla of life (Orlowski and Grinstein 2011; Donowitz et al., 2013; Fuster and Alexander 2014; Pedersen and Counillon, 2019). In mammals, 13 NHE paralogs have been identified thus far that participate in the regulation of cell volume, organellar and cell pH and contribute to extracellular volume and systemic pH homeostasis (Bobulescu et al., 2005).

**FIGURE 1:**
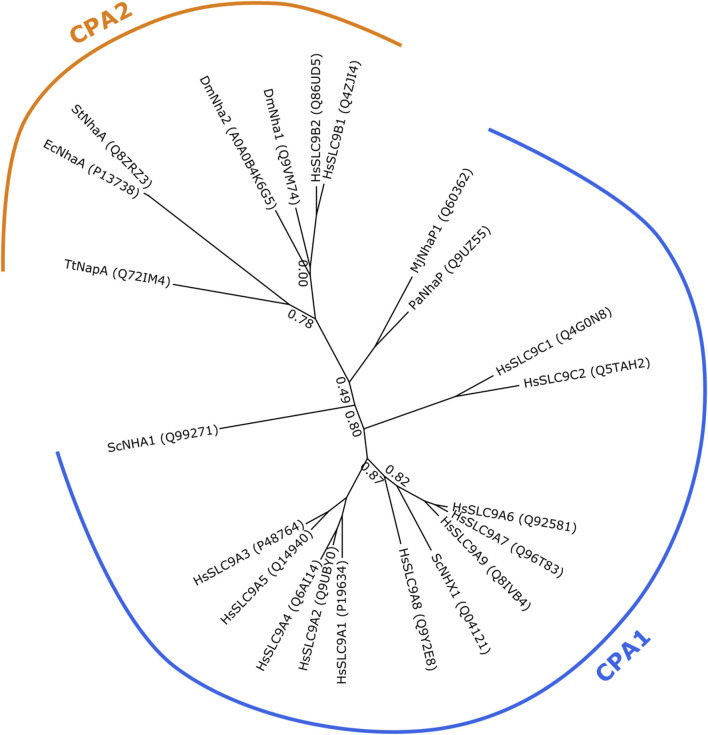
Phylogenetic tree showing evolutionary relatedness of Na^+^/H^+^ exchangers of the CPA1 and CPA2 clades. Protein sequences of human SLC9 members and other Na^+^/H^+^ exchangers of lower organisms with structural or functional relevance have been collected from UniProt (accession numbers shown in parentheses). Sequences were aligned using Clustal Omega 1.2.1 (Sievers et al., 2011; Sievers and Higgins, 2018) with five iterations and default settings. Smart Model Selection 1.8.4 (Lefort et al., 2017) and PhyML 3.3.20190909 (Guindon et al., 2010) were used to generate the phylogenetic trees, with 10 random starting trees and using the approximate likelihood ratio test aLRT method (Anisimova and Gascuel, 2006). The tree was visualized using TreeViewer 2.0.1 (https://treeviewer.org/). Branch support values below 0.9 are shown. CPA1 and CPA2 clades are marked. Dm, *Drosophila melanogaster*; Ec, *Escherichia coli*; Hs, *Homo sapiens*; Mj, *Methanocaldococcus jannaschii*; Pa, *Pyrococcus abyssi*; Sc, *Saccharomyces cerevisiae*; St, *Salmonella typhimurium*; Tt, *Thermus thermophilus*.

Various classifications for NHE’s exist, most notably the transporter classification database [www.tcdb.com; (Saier et al., 2006)] that classifies two NHE-families into the cation-proton antiporter (CPA) superfamily (subclassified into CPA1 and CPA2 clade, see Figure 1) and into the Na^+^-transporting carboxylic acid decarboxylase (NaT-DC) family. Using the classification set by the HUGO Gene Nomenclature Committee, mammalian NHEs are classified into the SLC9 gene family (solute carrier classification of transporters: http://slc.bioparadigms.org/)(Hediger et al., 2013). It consists of three proposed subgroups for the time being—the SLC9A subfamily (corresponding to the CPA1 cation/proton antiporter clade) is best described and consists of 9 plasma membrane or intracellular paralogs. The SLC9B subfamily (corresponding to the CPA2 clade) has two members, SLC9B1 and SLC9B2 (also known as NHA1 and NHA2 or NHEDC1 and NHEDC2, respectively), which have been much less studied. The SLC9C subfamily consists of two poorly characterized NHE’s, the sperm-specific SLC9C1 (sperm-NHE) and a putative NHE, SLC9C2 (Pedersen and Counillon 2019). The evolutionary relationships between human SLC9 proteins and similar non-mammalian proteins with structural or functional relevance are shown in Figure 1, which also illustrates the separation of the CPA1 and CPA2 subfamilies.

The existence of the members of the SLC9B subfamily was reported by Rao et al. (Brett et al., 2005). It has been proposed that they are more similar to prokaryotic NHEs compared to the other previously well-known mammalian NHEs, which mainly comprise SLC9A subfamily members (Brett et al., 2005). The orthologues of the SLC9B subfamily exist in most of metazoan genomes, including nematodes, flies, puffer fish, mice and humans. NHA1 is specifically expressed in testis, while NHA2 has been found to be fairly ubiquitously expressed (Ye et al., 2006; Xiang et al., 2007; Battaglino et al., 2008; Fuster et al., 2008). There is scant information on the physiological function of NHA1, and its role in human disease has not been explored. In contrast, significantly more studies have been carried out on the NHA2 paralog and various roles in human physiology and disease have been proposed.

This review encompasses the currently available knowledge on the structural, functional and pharmacological aspects of the SLC9B subfamily (SLC9B1-2), hereby referred to as NHA1 and NHA2, and focuses mainly on NHA2, the better-studied SLC9B paralog within this subfamily.

## 2 SLC9B1/NHA1

### 2.1 Introduction

NHA1 was first cloned from testis and proposed to be testis-specific by RT-PCR analysis. It has been mapped to human chromosome 4p24 and sequencing revealed a putative 515 amino acid residue protein with a molecular weight of about 56 kD by Western blotting (Ye et al., 2006). While localization studies indicated that NHA1 expression is specifically confined to sperm flagellum, NHA1 expression has also been observed in brain and endocrine organs (thyroid, parathyroid and pituitary glands) as well as in bones of humans, non-human primates and mice (Carithers et al., 2015; Holmes et al., 2016; Papatheodorou et al., 2019; Karlsson et al., 2021).

### 2.2 Structure

In contrast to the recently published NHA2 structure (Matsuoka et al., 2022), no structural information is currently available for NHA1. However, in view of the high sequence similarity of the two SLC9B paralogs, similarity is also assumed for the structural aspects. Using the published NHA2 structure as a template, we generated a model for the structure of NHA1 for the purpose of this review. Due to practical reasons, the comparison of structural aspects of NHA1 and NHA2 will be presented in the section focusing on the structural aspects of NHA2 (Section 3.2; Figures 3, 4).

### 2.3 Transport Characteristics

To date, no transport assay has been described for mammalian NHA1, and hence ion preference and transport kinetics remain unknown. Due to high sequence similarity with its paralogue NHA2 (discussed more in detail later in this manuscript), Na^+^/H^+^ exchange is most likely. This likelihood is further increased by functional data in sperm that strongly suggest Na^+^/H^+^ exchange by NHA1 (Balbach et al., 2020; Vyklicka and Lishko 2020; Kang et al., 2021; Wang et al., 2021). Interestingly, however, in one study, the Drosophila NHA1 orthologue (Nha1) expressed in Xenopus oocytes was proposed to function as a H^+^-Cl^−^ cotransporter instead of conducting Na^+^/H^+^ exchange (Chintapalli et al., 2015).

### 2.4 Physiological Function

#### 2.4.1 NHA1 in Sperm

The function of NHA1 has been best studied in mice sperm, where it has been shown to be expressed in sperm flagella and where it is believed to be important for sperm motility and thus male fertility. Besides NHA1 and sperm-NHE, the main NHEs present in sperm are NHE1, NHE5, and NHE8 (Klanke et al., 1995; Garcia and Meizel 1999; Goyal et al., 2003; Martins et al., 2014), although NHA2 has also been shown to be expressed. However, sperm-NHE (SLC9C1 or SLC9A10) and NHA1 (SLC9B1) are specifically expressed in the sperm flagellum, underscoring the importance of these two NHEs in sperm motility. This makes NHA1 the second most sperm-specific NHE next to sperm-NHE (SLC9C1), raising the question of whether the two NHEs present in spermatozoa are redundant. In fact, loss of either sperm-NHE alone, or of NHA1 and NHA2 in combination, has been shown to lead to infertility (whereas in the study by Chen et al. (2016), loss of only NHA1 or NHA2 was not sufficient for infertility but only resulted in reduced fertility). In detail, it was shown that when mouse spermatozoa were incubated with an NHA1 antiserum, spermatozoal intracellular pH and intracellular calcium levels were reduced, resulting in reduced sperm motility and reduced acrosome function. Similarly, a DNA vaccine against NHA1 reduced fertility in female mice, with sperm agglutination due to interaction of produced antibodies with SLC9B1 in the vagina (Wang et al., 2003; Liu et al., 2010; Chen et al., 2016). Recent work by another group using a different NHA1 knockout mouse also revealed that loss of NHA1 is sufficient to induce male infertility (Balbach et al., 2020). Additionally, genetic analysis in humans suggested disturbed SLC9B1 expression in men with infertility due to teratozoospermia. Another study also suggested differential methylation of the SLC9B1 promotor to affect sperm function (Kumar and James, 2015).

One might think that the loss of a single NHE in sperm should not affect fertility. However, control of pH is essential for sperm motility. Along the female reproductive tract, mammalian sperm are exposed to a wide pH range, with a gradual increase from the vagina (pH around 4.5 in humans) (Breckenridge et al., 1950) to the uterus, with highest pH found in the fallopian tubes (around 7.5). Since the optimal pH for sperm motility seems to be between 7 and 8, regulation of intracellular pH is of utmost importance for male fertility (Moghissi et al., 1964; Ng et al., 2018; Wang et al., 2021). As noted earlier, the exact contribution to pH regulation in sperm of the different NHEs present is not known and we suggest the interested reader to further consult reviews dedicated completely to the role of ion transport proteins in sperm for a more detailed description of proposed potential mechanisms (Vyklicka and Lishko 2020; Wang et al., 2021). However, since sperm-NHE and NHA1 appear to be the only members of this transporter family that are specifically expressed in sperm flagellum, one might assume that these transporters play an important role in flagellar function.

Important additional work underscores the importance of NHEs for male fertility. Namely, that the fertility of sperm-NHE-deficient mice can be partially rescued by alkalinization and completely rescued by addition of cAMP. This suggests that the loss of sperm-NHE (SLC9C1) is detrimental to fertility not due to changes in pH but due to a lack of cAMP as a result of inactive soluble adenylate cyclase (sAC), which is thought to form a complex with sperm-NHE (Wang et al., 2007). Interestingly, sea urchin sperm-NHE has been shown to mediate true Na^+^/H^+^ exchange but, unlike common SLC solute carriers, it is voltage-gated and directly modulated by cAMP, whereby the interaction of sea urchin sperm-NHE and sAC has not yet been fully elucidated (Windler et al., 2018). Whether similar mechanisms also apply to SLC9B1 remains to be determined.

The role of intracellular cAMP probably cannot be fully ignored, as knockout of sperm-NHE (SLC9C1) or NHA1/NHA2 has been shown to lead to reduced levels of intracellular cAMP, as well as a reduced protein expression of soluble adenylyl cyclase (sAC). This could be rescued by cAMP analogues, suggesting at least partial redundancy of NHEs to regulate cAMP levels (Wang et al., 2007; Chen et al., 2016).

Whether the functional redundancy of NHEs also holds true for spermatozoan pH control is less clear and needs further investigation. A recent study highlighted the role of NHA1 in Zona pellucida (ZP) induced pH modulation in sperm upon contact with the oocyte, an important part of the acrosome reaction necessary for the sperm to penetrate the zona pellucida to reach the oocyte. The same study also showed that NHA1-KO males are infertile, with a significantly reduced number of motile sperm and an altered sperm flagellar beat pattern. Interestingly, sAC function was unaffected in NHA1-KO sperm, suggesting a different mechanism of regulation by NHA1 and sperm-NHE (Balbach et al., 2020). We should be aware, though, that the significance of the acrosomal exocytosis induced by binding of sperm to the ZP for fertilization is not completely understood, as the acrosome reaction can already be induced in the oviduct before direct contact of the sperm with the zona pellucida (Baibakov et al., 2007; Muro et al., 2016).

Further studies utilizing patch-clamp recordings of mouse sperm also proposed functional coupling of NHE activity to the function of the sperm-specific K^+^ channel KSper, encoded by the *SLO3* gene (Schreiber et al., 1998; Zeng et al., 2011) and Ca^2+^ ion channels CatSper, encoded by the *CATSPER1* and *CATSPER2* genes (Quill et al., 2001; Ren et al., 2001), although specific inhibition of NHA1 and sperm-NHE hasn’t been performed to date to confirm this due to unavailability of specific inhibitors (Kang et al., 2021).

Furthermore, it remains to be seen whether the experiments performed with murine sperm can be replicated in human sperm, as it has been suggested that pH regulation in human sperm is different than in mice, and H^+^-extrusion may also be accomplished by voltage-gated H^+^-channels (Garcia and Meizel, 1999; Sasaki et al., 2006; Lishko et al., 2010; Miller et al., 2015).

Lastly, phylogenetic analysis suggested that the mammalian SLC9B1 gene, in contrast to the SLC9B2 gene (which seems to consistently exist since before vertebrate evolution), evolved following a gene duplication event of the SLC9B2 gene during marsupial evolution. This further questions whether SLC9B1 is essential for spermatozoan function as it evolved at a relatively late stage of evolution (Holmes et al., 2016).

### 2.5 Regulation

Little is known about the regulation of NHA1. The genes encoding NHA1 and NHA2 localize to the same chromosomal locus and are in fact adjacent to each other on mouse chromosome 3 and human chromosome 4q24, respectively, suggesting potential co-regulation (Rangwala et al., 2021). A recent study suggests that differential methylation of the NHA1 promoter may affect human male fertility (Kumar and James, 2015).

### 2.6 Drug Target Approaches

#### 2.6.1 Male Contraceptive Strategies

NHA1 is an obvious drug target for male fertility control since selective NHA1 inhibitors may be used as male contraceptives. However, before such approaches can be considered, the physiological function of NHA1 needs to be more clearly defined, especially its role in extragonadal tissues. Obviously, transport assays will also need to be developed to enable drug screening and characterization of identified compounds. Due to the high similarity between NHA1 and NHA2, the development of NHA1-specific inhibitors might be challenging, but is crucial to avoid unwanted off-target effects due to simultaneous inhibition of NHA2.

## 3 SLC9B2/NHA2

### 3.1 Introduction

Compared to NHA1, NHA2 has received much more attention since its initial discovery (Xiang et al., 2007; Battaglino et al., 2008; Fuster et al., 2008). Based on genomic localization, tissue distribution pattern and inhibitor characteristics (tolerance to amiloride but sensitivity to phloretin), NHA2 was proposed as a possible candidate for the elusive sodium-lithium countertransporter (SLC) (Xiang et al., 2007). An increased activity of this countertransporter in erythrocytes and fibroblasts is a heritable trait that has been linked to the pathogenesis of arterial hypertension and diabetes mellitus (Canessa et al., 1980; Mangili et al., 1988; Laurenzi et al., 1997; Strazzullo et al., 1998; Vaccaro et al., 2005).

### 3.2 Structure

#### 3.2.1 Structural Elements of CPA2 Transporters—Novel Information on NHA2

**FIGURE 2:**
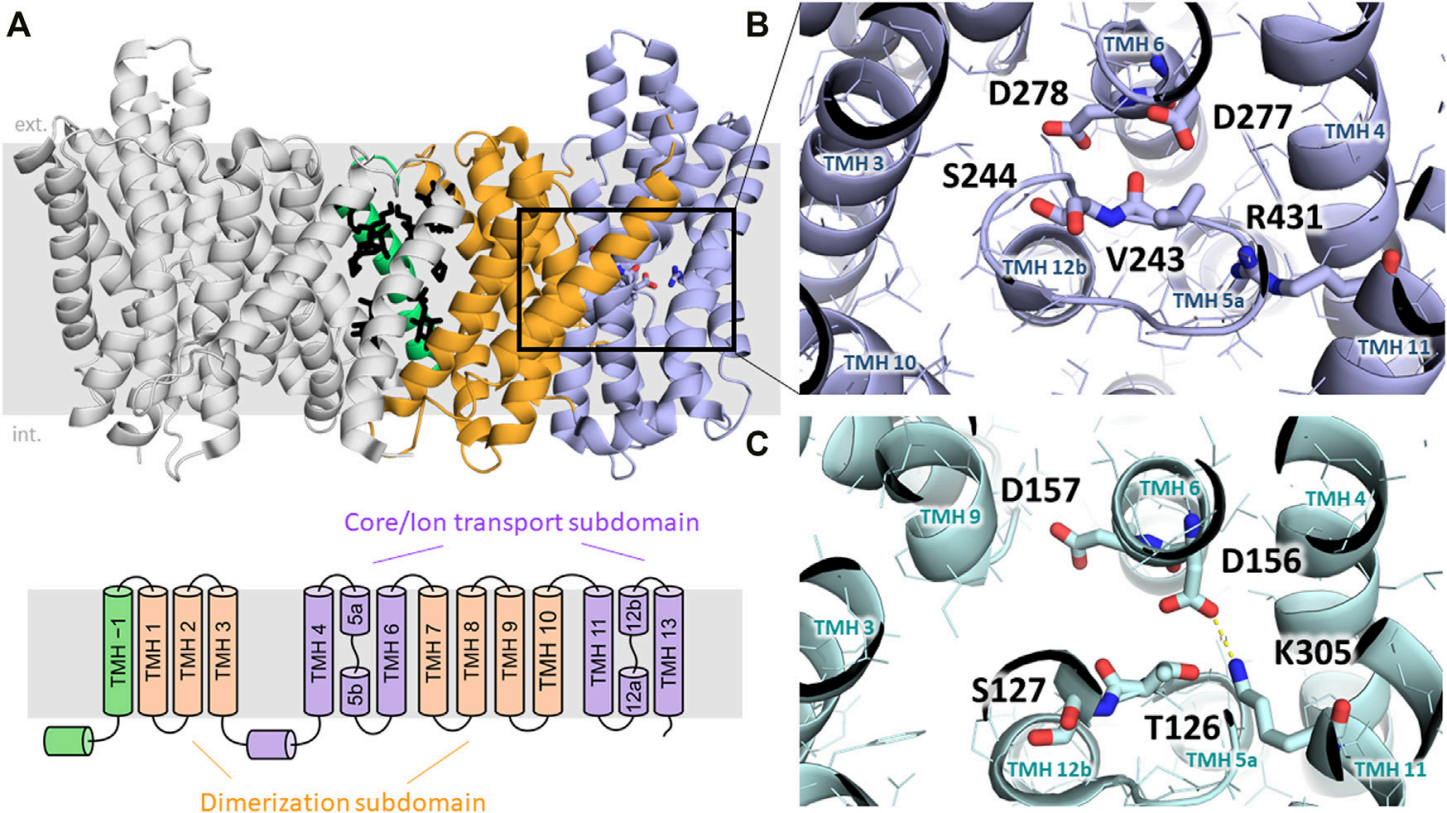
Structural elements of NHA transporters. **(A)** Dimeric structure of bison NHA2 (SLC9B2) in nanodiscs is shown with the two protomers in different colors (PDB ID: 7P1K). The core/ion transport subdomain, the dimerization domain and the “TM –1” region are colored in blue, orange and green, respectively. Lipids modeled based on electron density information are shown in black (Matsuoka et al., 2022). Below, the transmembrane architecture of NHA2 is shown. **(B)** Ion-binding site of bison NHA2 is shown with residues relevant for ion exchange highlighted. D278 and D277 are reported to bind Na^+^, and D278 was shown to be involved in H^+^ binding. S244 replaces the negative charge-compensation residue (see text). The backbone oxygen atom of V243 is predicted to interact with the bound Na^+^ ion (Matsuoka et al., 2022). The lack of a salt bridge between D277 and R431 is related to electroneutral transport (Matsuoka et al., 2022). **(C)** Ion-binding side of the electrogenic NapA exchanger is shown (PDB ID:4BWZ), residues analogous to those on panel B are marked (Lee et al., 2013). Electrogenicity is conferred by the H^+^-binding residue K305, which is locked in a salt bridge with D156 upon H^+^ binding (Lee et al., 2013).

Based on existing structural information, CPA superfamily proteins have been proposed to consist of 12–13 transmembrane helices (TMHs) (Hunte et al., 2005; Lee et al., 2013; Lee et al., 2014; Paulino et al., 2014; Wohlert et al., 2014; Winklemann et al., 2020; Dong et al., 2021), although recently a structure consisting of 14 TMHs has been shown for bison NHA2 (Matsuoka et al., 2022). The TMHs show an inverted repeat arrangement that is usually seen in secondary transporters (Forrest, 2015). The common functional domain consists of 12 TMHs that can be subdivided into a dimerization subdomain formed by TMHs 1–2 and 7–9, and a core or ion translocation domain formed by the six-helix bundle of TMHs 3–5 and 10–12 (Lee et al., 2013). In addition, several bacterial and archaeal CPA proteins, as well as mammalian NHEs contain an extra TMH at their N-termini, which takes part in forming dimer contacts between the two protomers and is usually referred to as “TM −1”. Furthermore, NHA2, according to the recently solved structure of bison NHA2 (Matsuoka et al., 2022), seems to contain another extra N-terminal TMH as part of its dimerization subdomain, which has also been referred to as “TM −1”, shifting the numbering of the remaining TMHs by one (Figure 2A).

Based on the comparison of inward- (cytoplasmic) open and outward-open conformations, CPA exchangers have been proposed to function using an elevator-like mechanism, where the dimerization subdomain forms an immobile scaffold relative to which the core subdomain can rotate along an axis parallel to the membrane plane, thus alternatingly exposing the central ion binding site towards either the cytoplasmic or the extracellular/periplasmic/luminal side of the membrane (Lee et al., 2013; Winklemann et al., 2020). This mechanism has also been observed in other, unrelated secondary carriers (Drew and Boudker, 2016). The recognition and binding of substrate ions takes place in the core subdomain, and is formed by two antiparallel transmembrane helices (TMH 5 and TMH 12 in NHA2) containing an unwound extended segment in the middle. These cross over so that two pockets are formed by the N-terminal ends of the half-helices TMH 5b and 12b, and the C-terminal ends of TMH 5a and 12a, respectively. Due to the opposite orientations of the aligned half-helices in each pair, their dipole moments form effective positively and negatively charged pockets, which are typically counterbalanced by the oppositely charged amino acid headgroups located in these pockets. This architecture is a hallmark of the “NhaA-fold”, and is ideally suited for binding small ions, while amino acid residues in this region can shape binding specificity and properties of transport (Lee et al., 2013). Interestingly, these charge compensating residues are not always conserved, and are sometimes replaced by non-charged residues, which is further elaborated below (Lee et al., 2013) (Figures 2B,C).

The first experimentally resolved structures of CPA transporters were those of NhaA from *E. coli* (Hunte et al., 2005; Lee et al., 2014) and NapA from *T. thermophilus* (Lee et al., 2013), both of which phylogenetically belong to the CPA2 clade and are thus related to mammalian NHA2 despite their low shared sequence identity (15%–21%). NhaA and NapA are both well characterized concerning their function and have been shown to perform electrogenic ion exchange with a stoichiometry of 1Na^+^:2H^+^, in contrast to CPA1 subfamily members, such as PaNhaP (Wohlert et al., 2014), MjNhaP1 (Paulino et al., 2014) or mammalian NHEs (Winklemann et al., 2020; Dong et al., 2021), which are electroneutral. Recently, also the atomic structure of bison NHA2 has been reported (Matsuoka et al., 2022). An overview of the evolutionary relationship between these proteins is shown in Figure 1.

The analysis of the proposed substrate-binding site of NhaA, NapA, bison NHA2 and a previously constructed structural model of human NHA2 (Schushan et al., 2010) helped identifying structural elements that shape ion binding and transport activity. A highly conserved “DD” motif consisting of two aspartic acid residues (D278 and D279 in human NHA2) is harbored in the center of the membrane-spanning core subdomain (Figures 2B,C). Those two aspartic acid residues have been proposed to be part of the ion-binding sites since they are essential for transport (Inoue et al., 1995; Hunte et al., 2005). The involvement of both D278 and D279 in binding Na^+^ is also supported by molecular dynamics simulations of NHA proteins (Coincon et al., 2016; Matsuoka et al., 2022). Initially, these two aspartate residues were thought to bind one proton each during the transport process of electrogenic NHA-like transporters, however, dimeric structures of NhaA (Lee et al., 2014) and NapA (Lee et al., 2013) have shown the first aspartic acid residue (corresponding to D278 in human NHA2) to be in a salt bridge with a charge-balancing lysine residue. Later molecular dynamics simulations (Huang et al., 2016) and following mutagenesis studies (Uzdavinys et al., 2017) have confirmed that this lysine residue is in fact the proton carrier (K300 in NhaA and K305 in NapA, see Figure 2C), and its replacement, such as in human NHA2, where the corresponding residue is an arginine, would abrogate the binding of the second proton and thus confer electroneutral transport activity. Nevertheless, creation of an electrogenic human NHA2 with the reverse mutant R432K was not successful, implying that other factors also play a role in shaping transport stoichiometry (Uzdavinys et al., 2017). Indeed, the subsequent bison NHA2 structure has shown that the residues corresponding to R432 and D278 in human NHA2 are about 6.5 Å away from each other and unable to form a salt bridge (Matsuoka et al., 2022). In addition, the p*K*_*a*_ value of arginine is generally significantly higher than that of lysine, which might prevent it from acting as a dynamic proton shuttle in the physiological pH range (Lee et al., 2014; Matsuoka et al., 2022). Interestingly, R432 (R431 in bison) does form a salt bridge, but with E215 instead (E214 in bison) according to the bison NHA2 structure, and swapping the two residues (as in the bison NHA2 E214R/R431E double mutant) retains function but shifts the specificity of the transporter from Na^+^ to Li^+^ (Matsuoka et al., 2022).

Interestingly, lipids seem to play a role in the stabilization of the dimerization subdomain, in particular the first TMH (“TM −1”) of bison NHA2. This region possibly plays a regulatory role through modulating the oligomerization state of NHA2, as either removal of this region or point mutations in this region can shift the protein towards a monomeric state, while in turn dimerization is required for optimal function (Matsuoka et al., 2022). Reconstitution in nanodiscs with pure yeast phosphatidylinositol (PI) caused a rearrangement of TM −1 and a more packed and compact dimer interface. In turn, PI and also phosphatidylinositol diphosphate (PIP_2_) have been shown to stabilize dimeric NHA2, even though high concentrations of PIP_2_ might disrupt the dimeric state. On one hand, this is in line with the observation that PI is enriched in intracellular organelles, which is the preferred localization of NHA2. On the other hand, the role of PIP_2_ in the regulation of NHA2, including the presence of the predicted extracellular binding site at the dimerization interface, still has to be clarified (Matsuoka et al., 2022). Overall, it is likely that lipid composition plays a role in regulating the function of NHA2 by affecting the stability of the dimer interface.

#### 3.2.2 Sequence Comparison Between Members of the CPA2 Clade Allows to Model Potential NHA1 Structure on the Structure of NHA2

**FIGURE 3:**
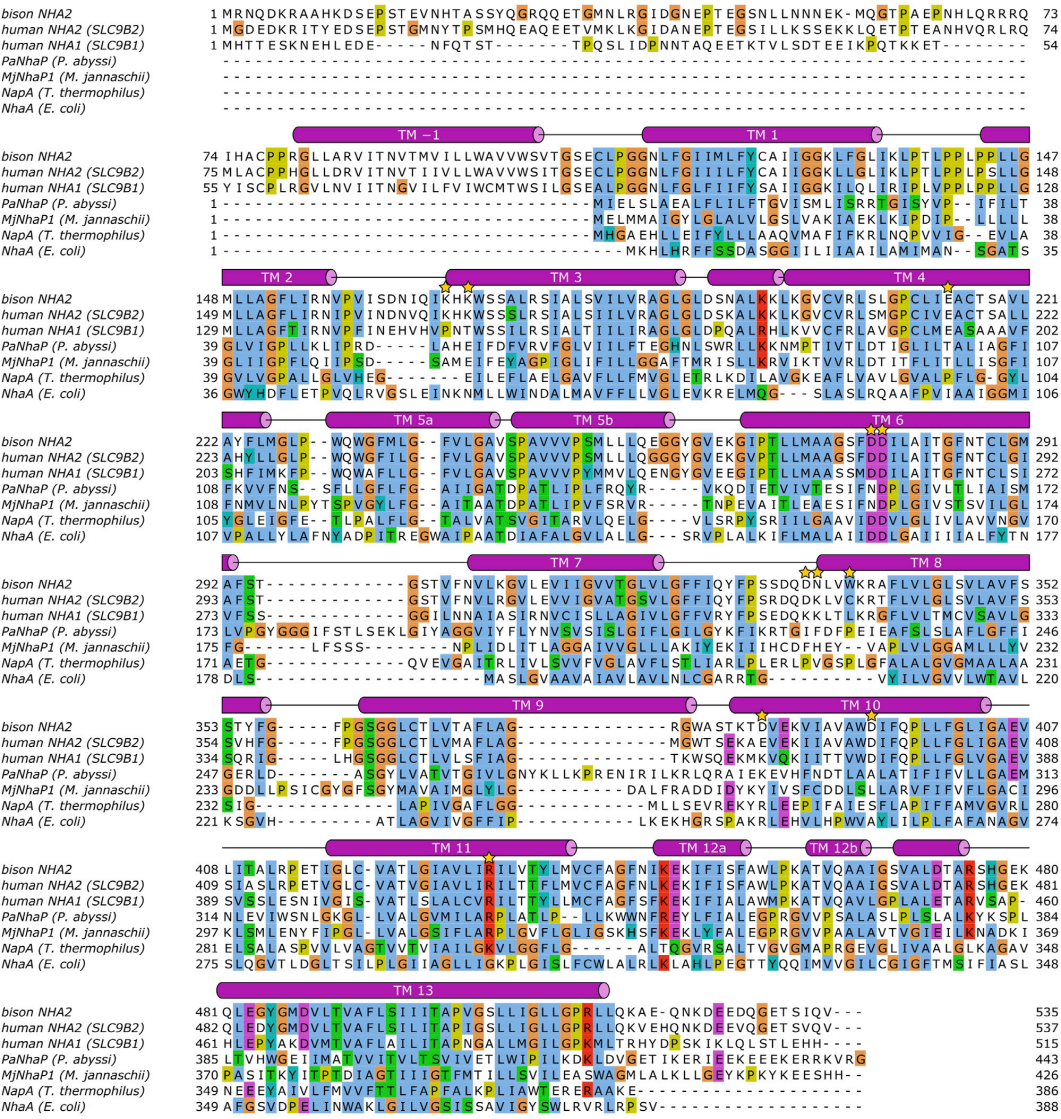
Sequence alignment of Na^+^/H^+^ exchangers. The alignment was calculated using ClustalW 2.1 (see text), and visualized using JalView 2.11.1.7 (Waterhouse et al., 2009). Helical segments shown as barrels have been added manually based on DSSP (Kabsch and Sander 1983) annotations of the bison NHA2 structure (Matsuoka et al., 2022). Transmembrane helices (TM) are labeled. The following residues are marked with a golden star: human NHA2 E215, D278, D279, R432; bison NHA2 K168, K170, W336; human NHA1 K313, K314, K364, D374.

**FIGURE 4:**
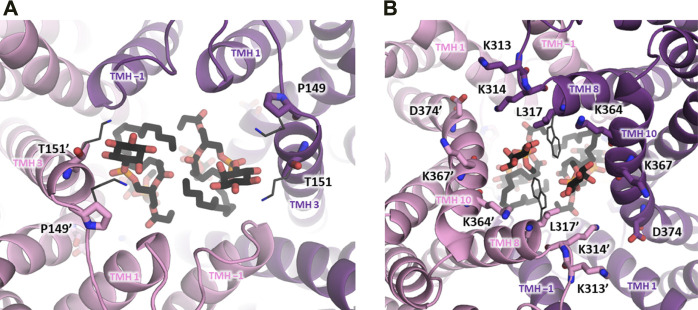
Dimerization subdomain of human NHA1. A homology-based model of human NHA1 is shown (see text), with the two protein subunits in purple and pink color. Lipid residues have been copied from the bison NHA2 structure (Matsuoka et al., 2022) and are shown in black stick representation for illustration. **(A)** View from the extracellular side, positions of lysine residues from bison NHA2 (K168, K170) suggested to interact with the phosphate groups of PIP_2_ are shown in dark grey thin stick representation, with their corresponding residues in human NHA1 labeled (P149, T151). **(B)** View from the cytoplasmic side, with putative lipid-interacting lysine residues shown in stick representation and labeled. Residue D374 can possibly form a salt bridge with K314’. The location of the W336 residue from bison NHA2, reported to interact with lipid inositol group, is shown in dark grey thin stick representation, with the corresponding L317 residue in human NHA1 labeled.

To facilitate comparison of NHA1 with other Na^+^/H^+^ exchangers with known structural information available, we have generated a multiple sequence alignment of the CPA transporter proteins mentioned in the paragraphs above using ClustalW 2.1 (Larkin et al., 2007) with default settings (Figure 3). Furthermore, in order to shed light on the structural aspects of NHA1-mediated ion exchange, we constructed a homology-based model of human NHA1 based on the recently published structure of bison NHA2, using the multiple alignment we generated (Figure 4). The modeled residues 60–497 of human NHA1 could be gaplessly aligned with the sequence of bison NHA2 (except residue K480 of bison NHA2), giving an overall sequence identity of 59.5% in the modeled region. In total, 50 models were built using MODELLER 9.21 (Webb and Sali 2016) with “slow” variable target function optimization (“library_schedule”), “very slow” molecular dynamics refinement, “max_var_iterations” set to 300, and two repeat cycles for the entire optimization. All other settings were left on default. Models were scored according to discrete optimized protein energy (DOPE) score (Shen and Sali 2006), and the model with the lowest score was selected as the best model. The best model was then fit on the bison NHA2 coordinates using PyMOL 2.1.1 (Schrödinger, LLC) to construct a dimer assembly of our human NHA1 model. Due to the high degree of sequence similarity and the essentially gapless alignment, the overall architecture of the protein as well as the ion-binding site was identical to that of bison NHA2. All residues in the binding site with functionally assigned roles are conserved, and we did not find residue changes that would create a charge imbalance and thus favor chloride (Cl^−^) ion binding near the ion-binding site, as suggested for the *Drosophila melanogaster* orthologue Nha1 previously (Chintapalli et al., 2015). In contrast, several residues that were proposed to play a role in PI or PIP_2_ binding in bison NHA2 (Matsuoka et al., 2022) are not conserved in human NHA1. In particular, on the extracellular side, K168 and K170, proposed to bind the negatively charged phosphate groups of PIP_2_, are replaced by P149 and T151 (Figure 4A), respectively; while on the cytoplasmic side, W336, interacting with the inositol group of bound lipids, corresponds to L317 in human NHA1. While several residues for PI or PIP_2_ binding are missing compared with bison NHA2, the cytoplasmic dimerization interface of human NHA1 seems to contain several lysine residues that are instead missing from bison NHA2, such as K364, K313, K314. One of these, K314, is in a good position to form an inter-subunit salt bridge with D374 of the other protomer. These differences suggest that NHA1 might have altered regulation of its function compared to NHA2, either through altered interactions with lipids, in particular phosphatidylinositols (PI or PIP_2_), or through the stability of the dimer interface itself. However, further mechanistic studies are required to validate these hypotheses.

### 3.3 Physiological Function and Regulation

#### 3.3.1 NHA2 in Cellular Sodium and pH Homeostasis

Since NHEs exchange cations for protons, they not only affect the intraluminal H^+^ concentration but also the luminal concentrations of Na^+^ and K^+^, depending on their ion selectivity. However, due to methodological hurdles, very little is known about the regulation of organellar Na^+^ and K^+^ by NHEs. In general, mammalian NHEs are believed to be electroneutral. However, detailed kinetic transport studies of intracellular NHE paralogs are lacking. In the case of electrogenic behaviors of certain intracellular NHE paralogs, mammalian NHEs could also affect the membrane potential (ΔΨ) of intracellular organelles, as is well documented in the case of electrogenic Cl^−^/H^+^ exchangers (Lippiat and Smith 2012). Currently available data suggest that NHA2 is an electroneutral NHE (Kondapalli et al., 2012; Chintapalli et al., 2015; Matsuoka et al., 2022).

Various studies have been performed to investigate the role of NHA2 on intracellular and/or intravesicular pH homeostasis, with conflicting results so far. In a pancreatic β–cell line, knockdown of NHA2 did not alter endosomal pH (Deisl et al., 2013). Similarly, endosomal and cytoplasmic pH measurements in NHA2-depleted or overexpressing cells did not show NHA2-dependent alterations in renal cell lines (Anderegg et al., 2021). Rao and colleagues suggested chemiosmotic coupling of NHA2 to the proton motive force generated by the V-type H^+^-ATPase at the plasma membrane of stably-NHA2-transfected MDCK cells, resulting in a virtual Na^+^ efflux pump (Kondapalli et al., 2012). Recent experiments with solid supported membrane (SSM)-based electrophysiology using proteoliposome-reconstituted bison NHA2 suggest electroneutral Na^+^/H^+^ exchange (Matsuoka et al., 2022). The results seem to recapitulate the experiments in cells, where Na^+^ export was driven by the inwardly directed pH gradient (which, in intracellular vesicles is generated by V-type H^+^-ATPases) (Kondapalli et al., 2012). However, high quality transport kinetics and functional studies of NHA2 in its native environment in the absence of overexpression are still lacking. Hence, the precise role of NHA2 in vesicular or intracellular Na^+^ and pH homeostasis is still unclear and needs further investigation.

#### 3.3.2 NHA2 Subcellular Localization

The subcellular localization of NHA2 remains controversial. Pham and colleagues first reported the specific localization of NHA2 in mitochondria in murine osteoclasts (Pham et al, 2007). But NHA2 does not seem to contain a mitochondrial targeting sequence (MTS) (Gyimesi and Hediger, 2020). Soon thereafter, overexpressed human NHA2 was shown to localize to the plasma membrane in yeast and the rat β-cell line INS-1, as well as to the apical membrane in polarized MDCK cells (Xiang et al., 2007). By immunostaining and subcellular fractionation assays using specific antibodies, endogenous NHA2 was found to co-localize with endosomal/lysosomal markers but not with mitochondrial markers in osteoclasts (Hofstetter et al., 2010). In line with these findings, NHA2 was also observed in endosomes of the murine β-cell Min6 (Deisl et al., 2013).

In the mouse kidney, NHA2 has been localized by immunofluorescence to the apical membrane of distal convoluted and connecting tubules (Kondapalli et al., 2017). NHA2 co-localized with the B1 subunit of the V-ATPase on the apical membrane of distal convoluted tubules (Kondapalli et al., 2017). In another recent attempt to clarify the subcellular localization of native NHA2 in the distal tubular cell line mpkDCT_4_ by sucrose gradient subcellular fractionation, endogenous NHA2 was present in the same fractions as the transferrin receptor, a marker of recycling endosomes, but was absent in plasma membrane fractions (Anderegg et al., 2021). Clearly, the subcellular localization of NHA2 and its function are not fully established and need further investigation.

#### 3.3.3 NHA2 in Osteoclasts

NHA2 is one of the most prominently upregulated transcripts during the RANKL (receptor-activator of the NF-κB ligand)-induced osteoclast differentiation and NHA2 silencing significantly inhibited osteoclast differentiation *in vitro* (Battaglino et al., 2008; Ha et al., 2008). To study the physiological role of NHA2, the Fuster laboratory generated two different strains of NHA2-deficient mice: a genetrap NHA2-deficient mouse and a targeted NHA2 knock-out mouse (deletion of exon 7 which encodes catalytically important amino acids). Interestingly, structural bone parameters quantified by high-resolution microcomputed tomography (μCT), bone morphology and osteoclast differentiation were not different between NHA2-deficient and wildtype (WT) mice. Similarly, *in vitro* RANKL-stimulation of osteoclast precursor cells isolated from NHA2-deficient mice did not lead to any alterations in osteoclast differentiation and resorptive activity compared to cells isolated from WT mice (Hofstetter et al., 2010; Deisl et al., 2013). These findings were later independently confirmed by another group using a different NHA2-deficient strain (Charles et al., 2012).

#### 3.3.4 NHA2 in the Endocrine Pancreas

NHA2 is highly expressed in human as well as rodent β–cells and β–cell lines (Xiang et al., 2007; Deisl et al., 2013). Knock-down of NHA2 in a murine β–cell line (Min6) reduced both glucose- and sulfonylurea-induced insulin secretion. This insulin secretion deficit could be rescued by overexpression of wild-type, but not functionally-dead human NHA2. Similar findings were obtained when islets of NHA2-deficient mice were studied, with loss of NHA2 leading to a ∼50% reduction of insulin secretion *in vitro*. *In vivo*, the insulin secretion deficit could be recapitulated, as NHA2-deficient mice displayed a pathological glucose tolerance with impaired insulin secretion, but normal peripheral insulin sensitivity.

NHA2 localizes to endosomes in β–cells, and loss of NHA2 attenuated clathrin-dependent endocytosis but did not alter the endosomal pH (Deisl et al., 2013). The detailed mechanism behind this observed impaired insulin secretion still remains to be elucidated. The current data suggest disrupted endo-exocytosis coupling, which has been shown to be important for proper β–cell function, as the underlying mechanism (Orci et al., 1973; MacDonald et al., 2005; Min et al., 2007; Kimura et al., 2008; Deisl et al., 2013).

Loss of NHA2 also exacerbated aging- and obesity-induced glucose intolerance in mice. Secretion of glucagon and the two major adipokines, leptin and adiponectin were not affected by loss of NHA2, again suggesting that the phenotype observed is mediated by the defective β–cell function (Deisl et al., 2016). In support of these findings, a single-nucleotide polymorphism in the *NHA2* gene (rs4699049) was recently discovered in a genome-wide association study as a new locus associated with type 2 diabetes (and renal function) in humans. (Liu et al., 2018; Hellwege et al., 2019)

#### 3.3.5 NHA2 in the Kidney

Similar to the role of NHA2 in the endocrine pancreas, NHA2 has been suggested to have a physiological role in renal tubular function and blood pressure homeostasis. Strong reasons for this proposition are the localization of the NHA*2* gene within a certain region of the fourth chromosome associated with the sodium-lithium countertransporter, its inhibitory characteristics (phloretin-sensitive and amiloride-insensitive), as well as distal tubular localization, a tubular segment important for sodium and blood pressure homeostasis (Canessa et al., 1980; Mangili et al., 1988; Carr et al., 1990; Meneton et al., 2004; Meneton et al., 2005; Ye et al., 2006; Xiang et al., 2007; Kondapalli et al., 2012). Localization of NHA2 to the distal nephron has already been reported upon initial description of the protein (Fuster et al., 2008), and recently further detailed and validated in mice, as well as humans (Kondapalli et al., 2017; Anderegg et al., 2021). Highest expression was found in distal convoluted tubules (DCT), with lower expression levels in connecting tubules (CNT) and both intercalated and principal cells of cortical collecting ducts (CCD). Interestingly, upregulation of NHA2 expression on mRNA and protein level in the kidney with a high sodium diet has been reported, suggesting a role of NHA2 in the pathogenesis of salt-sensitive hypertension (Fuster et al., 2008; Kondapalli et al., 2016; Anderegg et al., 2021) Similarly, the *Drosophila* orthologues Nha1 and Nha2 were shown to be induced by a high Na^+^ but not K^+^ diet (Chintapalli et al., 2015).

A recent publication shed some more light on the physiological function of NHA2 in the kidney, demonstrating that NHA2 KO mice exhibit a phenotype resembling the genetic condition in humans called Gitelman’s syndrome, resulting from loss-of function mutations or deletions of the apical thiazide-sensitive Na^+^/Cl^−^ cotransporter NCC (also known as SLC12A3) present in DCT cells, and characterized by hypovolemia with secondary hyperaldosteronism, reduced blood pressure, normocalcemic hypocalciuria and a blunted response to thiazide diuretics. Extensive *in vitro* and *in vivo* studies revealed that loss of NHA2 leads to a cell-autonomous, specific downregulation of the WNK4—SPAK—NCC axis in distal convoluted tubule tubular cells (Anderegg et al., 2021). The molecular basis for the changes observed are lower WNK4 levels, caused by increased WNK4 ubiquitylation due to reduced phosphorylation of KLHL3, an E3-RING ubiquitin ligase complex protein involved in WNK4 ubiquitylation, while phosphorylation of other cellular PKC and PKA substrates was not affected (McCormick and Ellison 2011; Castaneda-Bueno et al., 2012; Shibata et al., 2013; McCormick et al., 2014)). The loss of NHA2 neither affected levels of active, phosphorylated PKA or PKC isoforms responsible for KLHL3 phosphorylation, nor the abundance of the Ca^2+^ -sensitive phosphatase calcineurin, which was recently shown to be involved in KLHL3 dephosphorylation (Ishizawa et al., 2019). Similarly, quantification of intracellular Ca^2+^ levels, store-operated Ca^2+^ entry and membrane potential in NHA2 depleted cells were comparable to cells with normal NHA2 levels. Regarding experiments in β–cells, endosomal and cytoplasmic pH measurements in NHA2 depleted or overexpressing cells did not reveal changes. Hence, the molecular relationship between NHA2 and reduced KLHL3 phosphorylation, and thus the molecular basis of these findings, remains unknown at the moment. Although a kidney-specific NHA2 KO model was not used, results obtained with NHA2 KO and NHA2/NCC double KO mice and complementary *in vitro* data clearly point to an intrarenal, DCT-cell autonomous origin of the observed phenotype.

#### 3.3.6 NHA2 in the Pathogenesis of Autosomal-Dominant Polycystic Kidney Disease

Autosomal-dominant polycystic kidney disease (ADPKD) is the most frequent inherited kidney disease in humans, accounts for about 10% of patients with end-stage kidney disease and occurs in all ethnic groups (Chapman et al., 2015; Bargagli et al., 2020). The disease is characterized by progressive enlargement of the kidneys due to cyst growth, resulting in chronic flank pain, abdominal fullness and reduced quality of life (Rizk et al., 2009; Miskulin et al., 2014; Anderegg et al., 2020). Heterozygous mutations in the *PKD1* and *PKD2* genes encoding the transmembrane proteins polycystin-1 (PC1) and polycystin-2 (PC2), respectively, account for the overwhelming majority of ADPKD cases. However, the molecular mechanisms connecting the mutations in the genes to the pathogenesis of the disease are still poorly understood (Lanktree et al., 2021). Rao and colleagues examined publicly accessible mRNA expression data of kidneys from patients with ADPKD and found significant upregulation of NHA2 expression. Interestingly, upregulation of NHA2 expression positively correlated with cyst size and disease severity. Based on these results, the authors speculated that increased NHA2 expression may contribute to the pathogenesis of ADPKD (Song et al., 2009; Prasad et al., 2019).

This hypothesis was tested in an *in vitro* cystogenesis model employing Madin-Darby canine kidney (MDCK) cells that stably overexpress NHA2-GFP (Yang et al., 2008). In 3-dimensional cultures, NHA2 overexpressing cells produced significantly larger cysts compared to non-transfected controls, including multilayered and multiloculated cysts not observed in cultures with control cells. siRNA-mediated knock-down of NHA2 or addition of the non-specific NHA2 inhibitor phloretin to NHA2-GFP overexpressing MDCK cells attenuated cyst growth *in vitro*. Phloretin, however, had no effect on cytoplasmic pH. Using MDCK cells expressing wild-type and mutant PC1, the authors additionally demonstrated that an increase of intracellular Ca^2+^ due to store-dependent and store– independent Ca^2+^ entry, possibly caused by defective PC1-PC2 interaction, enhanced NHA2 expression through NFAT/Ca^2+^ signaling (Prasad et al., 2019). This finding is in line with previous reports describing NFAT-mediated induction of NHA2 transcript expression during osteoclast differentiation induced by RANKL (Pham et al., 2007; Battaglino et al., 2008).

Vasopressin-mediated V2 receptor signaling has been shown to significantly contribute to ADPKD pathogenesis as V2 receptor activation increases levels of cAMP, which then stimulates both fluid secretion and cell proliferation in ADPKD (Devuyst and Torres 2013). Rao and coworkers furthermore discovered that the two methylxanthines theophylline and caffeine (that inhibit phosphodiesterase and thereby increase intracellular cAMP levels) are associated with increased NHA2 expression. As basic mechanism it has been postulated that NHA2 may increase cyst fluid volume through its NHE-activity, or alternatively play a role in intracellular cAMP signaling, similar as has been shown for NHA1 in spermatozoa (Chen et al., 2016; Kang et al., 2021).

### 3.4 Drug Target Approaches

Compared to NHA1, NHA2 is not restricted to a specific organ, but has been found in all tissues examined (Xiang et al., 2007), which could hinder drug development due to off-target effects. However, in a given tissue, NHA2 is typically expressed in specific subsets of cells (e.g. osteoclasts of the bone (Hofstetter et al., 2010), β-cells of the endocrine pancreas (Deisl et al., 2013) or in distal convoluted tubules of the kidney (Fuster et al., 2008; Kondapalli et al., 2016), reducing the possibility of generalized cellular off-target effects.

The currently known main phenotype of loss of NHA2 being an insulin secretion deficit and reduced blood pressure mediated by reduced activation of the WNK-SPAK-NCC axis, drug development could focus on these two metabolic diseases, diabetes mellitus and arterial hypertension. Since loss of NHA2 leads to a reduced insulin secretion, an allosteric NHA2 activator would have to be developed to increase insulin secretion.

As loss of NHA2 reduces blood pressure, NHA2 inhibitors would very likely lead to a similar effect. Since the effect of reduced blood pressure is a consequence of reduced activation of the WNK-SPAK-NCC axis and there have been drugs blocking NCC since decades (thiazide diuretics such as hydrochlorothiazide or metolazone), the additional benefit of another inhibitor of this pathway would need to be further elaborated. NHA2 inhibitors might be beneficial in genetic syndromes of arterial hypertension (e.g., Gordon’s syndrome) that lead to increased abundance and activity of WNK-kinases (and subsequently the SPAK-NCC axis) to directly inhibit the increase in WNK-kinase activity before the entire cascade is stimulated. This would allow a more specific approach to block overactive WNK-kinase and potentially reduce side-effects, compared to the blockage of NCC at the end of the activation cascade by thiazides. Similar efforts, albeit at an intermediate level, are already underway with the development and in some case already patenting of inhibitors of the SPAK-kinases, their sister kinase OSR1 or the SPAK-WNK interaction to be used as antihypertensive agents, but also as neuroprotective agents after a stroke (Kikuchi et al., 2015; AlAmri et al., 2017a; AlAmri et al., 2017b; Zhang et al., 2020; Fujii et al., 2021; Josiah et al., 2021).

Another potentially promising drug targeting approach would be the development of NHA2 inhibitors for the treatment of ADPKD. Currently, only the V2 receptor antagonist tolvaptan is approved for the treatment of ADPKD (Torres et al., 2012; Torres et al., 2017; Torres et al., 2020). In addition, tolvaptan’s side effect profile prevents its use in many ADPKD patients and the efficacy of the therapy is limited (Anderegg et al., 2020). Hence, there is a dire need for additional therapies to treat ADPKD. Despite many open questions about the basic mechanisms of action and the current lack of *in vivo* studies, the available data indicate that NHA2 may play a distinct role in the pathogenesis of ADPKD. As such, NHA2 represents a promising druggable target for the treatment of ADPKD that merits further exploitation.

## 4 Discussion

The phenotypes observed upon loss of NHA1 and NHA2 in various organs and mechanistic studies addressing pH homeostasis and/or transport function of NHA1 and NHA2 open discussions on the general physiological roles of intracellular NHEs or SLC9B subfamily members. The primary localization likely in intracellular compartments (Battaglino et al., 2008; Fuster et al., 2008; Hofstetter et al., 2010; Deisl et al., 2013; Anderegg et al., 2021) and observed changes in endocytosis (Deisl et al., 2013) suggest a role of the SLC9B subfamily members as intracellular NHEs involved in the regulation of intracellular vesicular transport as well as vesicular electrolyte and pH regulation. Clearly, additional detailed mechanistic studies are needed to advance our understanding of the role of the SLC9B subfamily in regulating distribution of electrolytes and pH homeostasis of intracellular compartments.

### 4.1 Functional Redundancy and Compensatory Upregulation of Members of the SLC9B Family

A study focused on the orthologues of NHA1 and NHA2 in Drosophila has proposed that insect Nha1 (which is more similar in sequence identity to human NHA2), as well as insect Nha2, are essential for fluid secretion in epithelia, including Malpighian tubules. Furthermore, Drosophila Nha1 and Nha2 seem to compensate for their losses through mutually compensating upregulation. Furthermore, Chintapalli et al. (2015) found NHAs to be necessary for sodium salt stress and essential for fruit fly viability (Day et al., 2008).

It could therefore be speculated that a similar compensatory upregulation of NHA1 and/or NHA2 upon loss of the other isoform could also occur in mammalian SLC9B isoforms. This could explain the minor phenotype observed in bone for NHA2 KO mice and the subfertility observed in NHA1 or NHA2 KO mice, with infertility only upon double KO, although this has recently been challenged (Chen et al., 2016; Balbach et al., 2020).

The possibility of functional redundancy of members of the SLC9B subfamily on the one hand and other endosomal NHE paralogs on the other hand can be speculated from various experimental evidence. Possible functional redundancy has been proposed to explain the lack of a bone phenotype of NHA2 KO animals, especially as NHA2 is one of the most highly upregulated proteins in osteoclasts and has been widely used as a marker for osteoclast formation (Battaglino et al., 2008; Hofstetter et al., 2010; Charles et al., 2012). The obvious candidate for functional compensation would be the paralogue NHA1. This could be further studied by analyzing the bone phenotype of NHA1/NHA2 double KO mice. NHA1/NHA2 double KO mice have been previously reported to exhibit male infertility. The bone phenotype, however, has not been studied. It should be noted, however, that the authors reported the creation of this line by crossing NHA1 and NHA2 KO mice. Given that NHA1 and NHA2 are positioned directly next to each other on chromosome 3 (and equivalently in humans on chromosome 4q24) (Rangwala et al., 2021), the generation of such a line by simple cross-breeding is doubtful and hence the validity of the findings reported with this mouse line is unclear.

### 4.2 The Precise Role of NHA2 in Endocytosis and Intracellular Ion and pH Homeostasis Requires Further Investigation

Thus far, no change in cytosolic or endosomal pH has been reliably demonstrated upon loss or overexpression of NHA2 (Deisl et al., 2013; Anderegg et al., 2021). While it may be that the main function of NHA2 is not pH regulation, subtle changes in pH might also have been missed due to methodological constraints, as measurement of pH was only conducted in a subset of endosomes. Likewise, valid measurements of intra-vesicular electrolyte content in native cellular models (without overexpression of NHA2) haven’t been performed so far.

Similarly, our knowledge of the expression and intracellular localization of native NHA2 is considerably lacking in certainty and detail again due to methodological limitations in localization of native NHA2. The cellular localization furthermore differs depending on the subset of cells, with clearly plasma membrane expression in osteoclasts (noting that the basolateral plasma membrane of the osteoclast can be functionally compared to a lysosomal membrane), but possible endosomal expression in pancreatic and kidney cells.

Correspondingly, the role of NHA2 in regulating endocytosis remains to be further elucidated. While loss of NHA2 has been shown to reduce clathrin-dependent endocytosis, it has not been possible to elucidate further mechanistic aspects. Another aspect concerning endosomal function is functional redundancy of endosomal NHEs in general. Detailed studies on the endosomal electrolyte and pH homeostasis with high spatial and temporal resolution should yield more information on the role of the SLC9B family in the cell. Furthermore, progress in the field would be greatly aided by the availability of specific inhibitors (and ideally allosteric activators) for NHA1 and NHA2, highly sensitive and specific antibodies suitable for immunoblotting and microscopy and high-resolution kinetic membrane transport assays.

### 4.3 Potential Role of NHA2 in the Brain and Other Organs With High Expression

In microarray and transcriptomics datasets, the highest expression of NHA2 is found in the liver. Very high expression of NHA2 is also found in almost all parts of the brain, without significant regional specificity. Furthermore, NHA2 is highly expressed in the upper gastrointestinal tract and female reproductive organs (Fuster et al., 2008; Uhlen et al., 2015; Sjostedt et al., 2020). NHA2 KO mice have no apparent hepatic, gastrointestinal or neurological phenotype, but detailed studies have not been reported. Given the known association of other endosomal NHEs (NHE6 and NHE9) with neurological diseases (e.g. NHE6 for Christianson syndrome and NHE9 for autism and ADHD) (Kondapalli et al., 2013; Ouyang et al., 2013; Pescosolido et al., 2014; Schwede et al., 2014; Prasad et al., 2017; Gao et al., 2019; Lee et al., 2021; Pescosolido et al., 2021) and potential functional redundancy of endosomal NHEs, it is possible that NHA2 also has an important, not yet understood, function in the brain or other organs that have not yet been studied in detail.

In summary, while recent efforts have greatly extended the knowledge on transport characteristics, physiological function and structural aspects of the SLC9B subfamily, there is still a considerable lack of our understanding of the precise molecular functions of NHA1 and NHA2.
